# Telmisartan protects chronic intermittent hypoxic mice via modulating cardiac renin-angiotensin system activity

**DOI:** 10.1186/s12872-018-0875-4

**Published:** 2018-07-03

**Authors:** Wanyu Wang, Ailing Song, Yiming Zeng, Xiaoyang Chen, Yixiang Zhang, Yonghong Shi, Yihua Lin, Wen Luo

**Affiliations:** 1 Pneumology Department of the First Affiliated Hospital of XiaMen University, The first clinical medical college of Fujian Medical University Teaching hospital of Fujian Medical University, Xiamen, China; 2 Pneumology Department of Wuxi Branch of Rijin Hospital affiliated to Shanghai jiaotong university medical college, Wuxi, China; 3 Department of Pulmonary and Critical Care Medicine of the Second Affiliated Hospital of Fujian Medical University, Sleep and Breathing Disorders Research Institute of Fujian Medical University, No.34 Zhongshan North Road, Licheng District, Quanzhou, 362000 Fujian China

**Keywords:** Chronic intermittent hypoxia, Sleep apnea syndromes, Telmisartin, ACE, ACE 2, Ang II, Myocardial apoptosis; myocardial cell ultrastructure

## Abstract

**Background:**

To explore the effects of chronic intermittent hypoxia (CIH), which mimics sleep apnea syndrome, on the cardiac renin angiotensin system (RAS), and to investigate the cardiac protection of an angiotensin receptor blocker (ARB)telmisartan (TERT) against CIH.

**Methods:**

32 healthy male C57B6J mice were randomly divided into CIH, ARB, blank and air control groups. CIH lasted for 12 weeks. Cardiac angiotensin converting enzyme (ACE), angiotensin converting enzyme 2 (ACE 2) and angiotensin II (Ang II) were evaluated by immunohistochemistry. Myocardial apoptosis were assessed by TUNEL assay and myocardial cell ultrastructure were observed under transmission electron microscope.

**Results:**

Cardiac ACE expression was higher in the CIH group than in blank and air control groups, which was decreased with TERT treatment. TERT treatment elevated the expression of cardiac ACE 2 and Ang II compared with CIH group. Myocardial cell and capillary endothelial cell apoptosis, mitochondrial injury were most severe in CIH groups, which were mitigated with TERT treatment.

**Conclusions:**

CIH changes the expression of cardiac ACE, ACE2 and Ang II, which may cause myocardial damage. TERT protects mice from CIH-linked cardiac damage via modulating the activity of RAS in the hearts.

## Background

It has been well documented that the incidence of sleep apnea syndromes (SAS) is high in the general population and that SAS may lead to cardiovascular disease (CVDs) [1]. Chronic intermittent hypoxia (CIH) is one of the distinctive pathophysiological features of SAS, and is implicated in the development of SAS-associated CVDs. One of the mechanisms by which CIH promotes CVDs is the activation of the renin-angiotensin system (RAS) [2], a well-known contributor to the pathogenesis of CVDs. However, a key component of RAS, angiotensin converting enzyme 2 (ACE 2), inhibits RAS and exhibits vasodilatatory and anti-proliferative functions [3]. Currently, the effects of ACE 2 on the cardiovascular system have been investigated in diabetic animal models. However, the expression and function of ACE 2 in the hearts of CIH animals has not, to date, been explored. Previously, we reported that we successfully generated a CIH mouse model and studied the potential mechanisms by which CIH impaired cardiovascular function [4]. In our published work, we also investigated the protective effects of an angiotensin receptor blocker (ARB), telmisartan (TERT), that both has vasodilatory and anti-oxidative activity and is widely used clinically as an antihypertensive [5–7]. We found that CIH increased oxidative stress in the mouse hearts, as evidenced by increased levels of 8-hydroxy-2′-deoxyguanosine/8-hydroxyguanosine (8-OHdG/8-OHG), malondialdehyde (MDA), and NADPH oxydase p47, which were suppressed by TERT treatment [4]. In the present study, we investigated the mechanisms by which CIH affects the RAS. Furthermore, we explored whether TERT could protect against CIH-induced cardiac damage via modulating the activity of the RAS. Our goal was to elucidate the mechanisms by which TERT, and more generally, ARB, may act in the treatment of SAS.

## Methods

The experimental protocol was approved by Laboratory Animal Ethics Committee of the First Affiliated Hospital of Xiamen University. CIH, ARB treatment, blank control and air control groups, as described previously [4].

A total of 32 healthy male C57B6J mice, with a body weight between 20 and 25 g, were provided by the Laboratory Animals Center, Chinese Academy of Sciences, Shanghai (License number: SYXK (Fujian) 2008–0001). A self-made intermittent hypoxia box, an air control box, and a gas control system to control the gas cycle from oxygen and nitrogen gas to air were used in this study. The S-450 oxygen-detection alarm was purchased from American IST-AIM Company with a sensitivity of 0.1%. Medically compressed oxygen with a concentration higher than 99%, compressed nitrogen gas with a concentration higher than 99%, Atman-6500 air pump. The CIH profiles consisted of alternating room air (21% oxygen) and 5.5% oxygen every 120 s. Telmisartan (TERT) tablets (Micardis) were provided by the Shanghai Boehringer Ingelheim Company. Rabbit-anti-mouse antibodies against ACE, ACE 2 and Ang II were obtained from the Wuhan Boster Company. PV9001 kit and diaminobenzidine (DAB) coloring reagent kit were obtained from Beijing Zhong Shan Golden Bridge Inc. In Situ Cell Death Detection Kit (11684817910) was obtained from Roche. JEM-2100HC transmission electron microscope with a charge-coupled device camera (JEM-2100HC) operating at 120 Kv was purchased from Japan Electronics Co, Ltd.

### CIH and ARB treatment model

The experimental mice were randomly divided into four groups (8 per group): CIH, ARB treatment, blank control and air control groups. An intermittent hypoxia system was used to generate the CIH model. Briefly, the self-made plexiglass box was constructed with an aperture closed by hyaline film and bilateral wells. The production well contained a one-way flap. The gas inside the box had a constant flow to avoid carbon dioxide retention. The gas control system contained a micro-computer chip, control procedures, relays, solenoid valves and monitors. Each gas supply had an individual air ventilation pipe, all of which were controlled by solenoid valve switches. The oxygen concentrations inside the box were tightly controlled. Each episodic hypoxia cycle time was 2 min (i.e. nitrogen 30 s, resting 30 s, oxygen 20 s and air 40 s). Each gas insufflation was shown on the control program monitor. The oxygen concentration in the box was monitored by the oxygen-detecting alarm.

Mice in CIH and ARB groups were placed in the intermittent hypoxia box. Intermittent hypoxia lasted for 12 weeks with 8 h per day. After eight weeks of intermittent hypoxia, the mice in ARB group were intragastrically administered TERT solution, which contained 0.2 mL normal saline with the TERT concentration 10 mg/kg/d [8], once per day for 4 weeks. In the air control group, the control system and plexiglass box used was the same as that of the CIH and ARB groups in order to reproduce the same environment. The cycle was similar to the one used in CIH and ARB groups in order to produce the same noise but only air was used. The blank control group had no any gaseous interference.

After 12 weeks, mice were sacrificed by heart dissection under anesthesia. Mice were fasted overnight followed by anesthesia with an intraperitoneal injection of 3% phenobarbital (30 mg/kg), all mice were weighted. Blood samples were collected through a direct cardiac puncture, and were centrifuged at 3000 g for 15 min at 4 °C while the supernatants (serum) were collected and stored at − 80 °C for further analysis. Mice were vascularly perfused through the heart with cold 100 mM phosphate buffer (pH 7.4). Tissues were immediately removed and the heart was dissected, and cut into small pieces within 1 mm cubes. The other part of the apex of the hearts was fixed in 10% neutral buffered formalin, stored in 70% ethanol, paraffin embedded and sectioned for subsequent histological analysis and TUNEL assay.

### Determination of cardiac ACE, ACE 2 and Ang II, TUNEL assay, transmission electron microscope

Immunohistochemistry with the PV9001/DAB two stage method was used to detect the expression of ACE, ACE 2 and Ang II. The primary antibody was used at a concentration of 1:50. All positive staining was brown-yellow. The average optical density values were calculated by IPP 6.0 software. After dewaxing and rehydration, sections were incubating in proteinase K (400 μg/mL) for 10 min at 37 °C. TUNEL staining was then performed using In Situ Cell Death Detection Kit (Roche, 11,684,817,910) according to the instructions. After DAB substrate detection, sections were counterstaining with hematoxylin, mounted under glass coverslip and analyzed under light microscope. TUNEL-positive cardiomyocytes in each group were carefully evaluated under double-blinded conditions. Ten high-power fields (× 400) were randomly selected and scored, and the percentage of TUNEL-positive cells was determined by dividing the numbers of positive-staining nuclei by the numbers of total nuclei of the cells. The samples for transmission electron microscope were fixed with 2% glutaraldehyde in 0.1 m PBS overnight at 4 °C. After brief washing with PBS, the samples were post-fixed with 2% osmium tetroxide and 0.5% potassium ferricyanide in 25 mM cacodylate buffer at 22 °C, followed by dehydration, infiltration and embedding in Spurr’s resin. Thin sections (70 nm) were made and post-stained with uranyl acetate and lead citrate. Samples were viewed under transmission electron microscope. Images were captured at magnifications of 15,000~ 40,000.

### Statistical analysis

All of data are presented as the means ± standard error of the mean unless specified otherwise. One-way analysis of variance (ANOVA) followed by the Bonferroni post-hoc test was used to examine statistical comparisons of the mouse cardiac ACE2, and non-parametric Wilcoxon rank sum test was used to determine the statistical significance of cardiac ACE and Ang II data, the rate of apoptotic cells among these groups. The *P* values were calculated from two-tailed test. *P* < 0.05 was considered significant. Statistical analyses were performed using SPSS 20.0.

## Results

### TERT decreased the cardiac expression of ACE but increased the expression of ACE 2 and Ang II in CIH

**Fig. 1:**
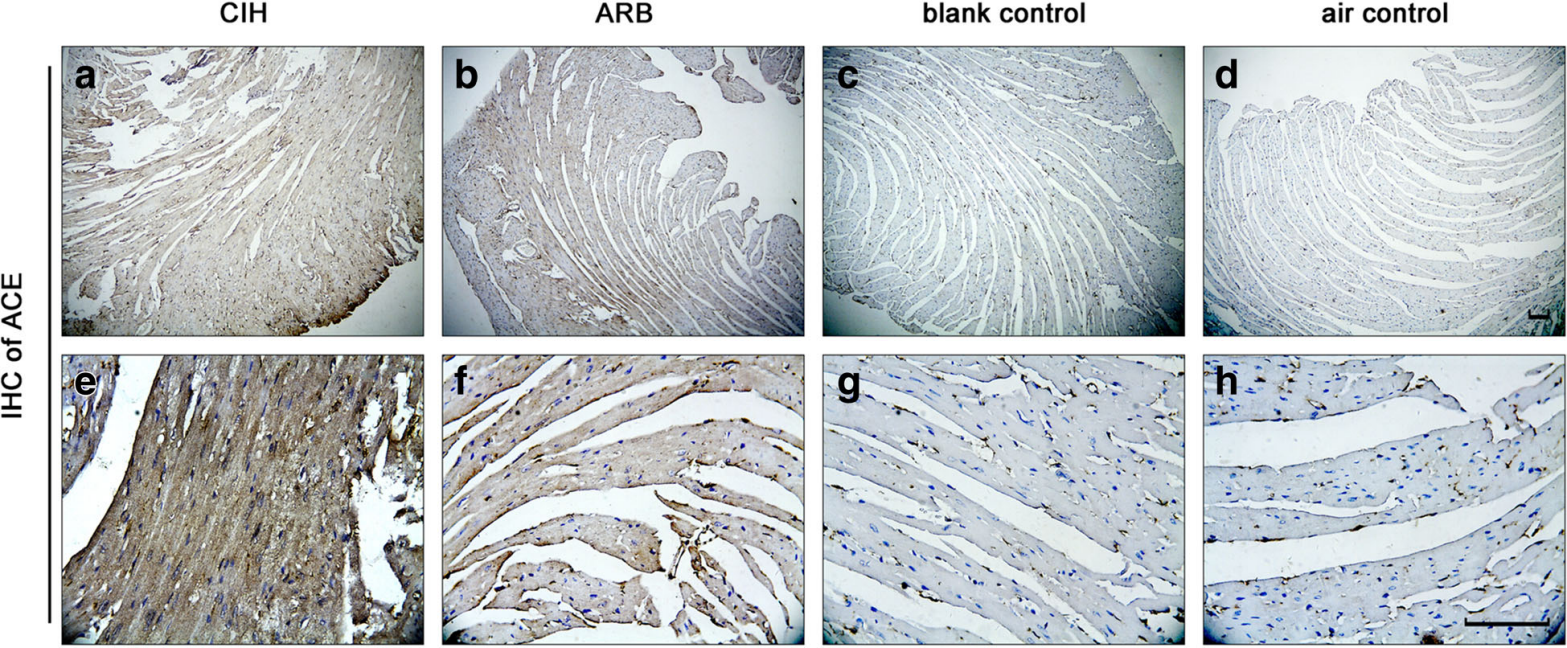
ACE staining in myocardial cells of mice from four experimental groups. **a** & **b**: CIH group; **c** & **d**: ARB group; **e** & **f**:blank control group; **g** & **h**: air control group. Immunohistochemistry was performed on the cardiac apex sections as described in Materials and methods. CIH and ARB groups showed highest ACE staining in CIH group followed by ARB group compared with the two control groups although this reduction did not reach statistical significance. Magnification: **a**, **c**, **e** and **g**: 100×; **b**, **d**, **f** and **h**: 400×. scale bar,100 μm

**Fig. 2:**
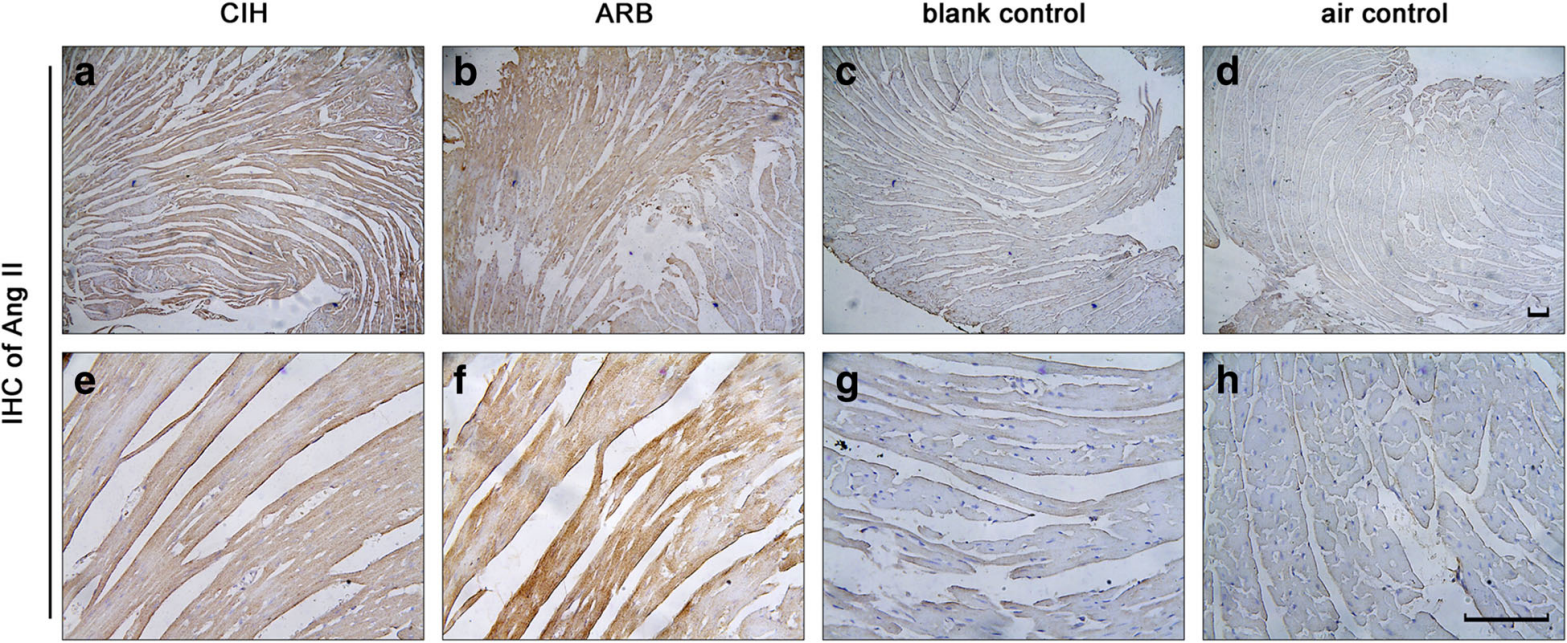
Ang II staining in myocardial cells of mice from four experimental groups. **a** & **b**: CIH group; **c** & **d**:ARB group; **e** & **f**:blank control group; **g** & **h**: air control group. Immunohistochemistry was performed on the cardiac apex sections as described in Materials and methods. The expression level of cardiac AngII was highest in ARB group followed by CIH group compared with the two control groups. Magnification: **a**, **c**, **e** and **g**: 100×; **b**, **d**, **f** and **h**: 400×. scale bar, 100 μm

**Fig. 3:**
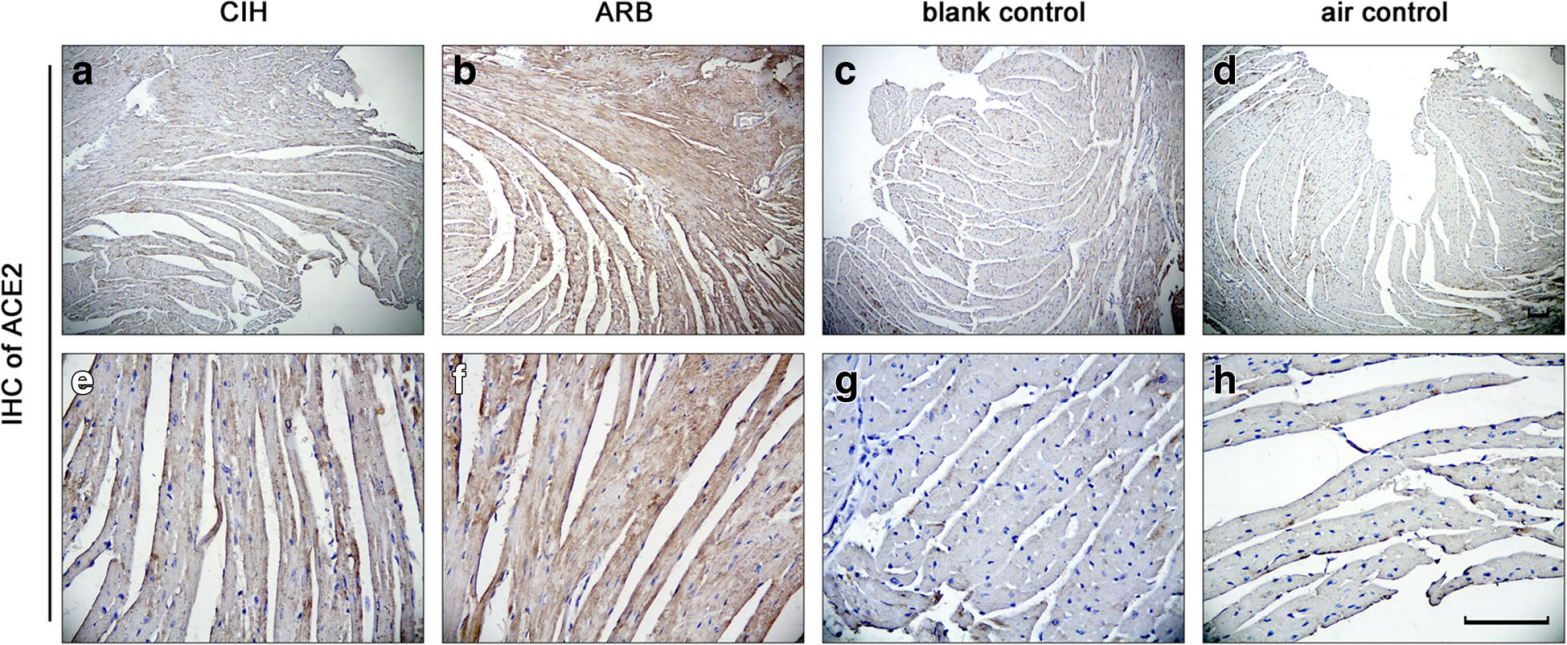
ACE 2 staining in myocardial cells of mice from four experimental groups. **a** & **b**: CIH group; **c** & **d**:ARB group; **e** & **f**:blank control group; **g** & **h**: air control group. Immunohistochemistry was performed on the cardiac apex sections as described in Materials and methods. The expression level of cardiac ACE 2 was highest in ARB group followed by CIH group compared with the two control groups. Magnification: **a**, **c**, **e** and **g**: 100×; **b**, **d**, **f** and **h**: 400×. scale bar,100 μm

Immunohistochemistry was used to determine the expression levels of ACE, ACE 2, and Ang II in the apex of mouse hearts obtained from different experimental groups, which are quantified and presented in Table 1. CIH and ARB groups showed stronger ACE staining (Fig. 1a-d) compared with the two control groups (Fig. 1e-h). Similarly, the expression level of cardiac ACE was significantly higher in the CIH group compared to that in the two control groups (*p < 0.001, vs. two controls. Table 1). TERT treatment reduced the level of ACE, although this reduction did not reach statistical significance compared with the CIH group. No significant difference in ACE expression was found between blank control and air control groups. In the myocardium of the apex of the heart, Ang II staining was mainly localized in the cytoplasm (Fig. 2a-h), and its expression was higher in CIH group than in blank and air control groups (^#^*p* < 0.05 vs two control groups). However, TERT treatment significantly increased Ang II levels in the heart compared with other experimental groups. (^&^p < 0.05 vs. CIH). No significant difference in cardiac AngII expression was found between blank control and air control groups. ACE 2 positive cells showed a similar cytoplasmic staining pattern in cardiac samples obtained from each experimental group (Fig. 3a-h). The expression level of cardiac ACE 2 was higher in the CIH group than in blank and air control groups (+p < 0.05, Table 1), however, TERT treatment significantly increased ACE 2 expression in cardiac samples (^$^*P* < 0.001 vs. CIH). There were no significant differences between blank control and air control groups. Collectively, we conclude that TERT decreased the cardiac expression of ACE but increased the expression of ACE 2 and Ang II in CIH mouse hearts.

**Table 1:** TERT decreased the cardiac expression of ACE but increased the expression of ACE 2 and Ang II in CIH

Group	n	Cardiac ACE	Cardiac Ang II	Cardiac ACE 2	Apoptotic rate of myocardial cells by TUNEL assay (%)
CIH group	8	0.034900 ± 0.0130^a^	0.095922 ± 0.0328^d^	0.052200 ± 0.0192^b^	53.23 ± 5.292^f^
ARB treatment group	8	0.025100 ± 0.0061^a^	0.149025 ± 0.0217^d c^	0.072850 ± 0.0175^b c^	23.32 ± 2.827
Blank control group	8	0.004738 ± 0.0026	0.015019 ± 0.0070	0.017488 ± 0.0131	22.39 ± 3.715
Air control group	8	0.005238 ± 0.0026	0.020684 ± 0.0107	0.029313 ± 0.0136	23.72 ± 2.857

^a^*p* < 0.001 vs. blank and air control groups

^b^*p* < 0.05 vs. blank and air control groups

^c^*p* < 0.001 vs. CIH

^d^*p* < 0.05 vs. blank and air control groups

^e^*p* < 0.05 vs. CIH

^f^*p* < 0.001 vs. the other three experimental groups

### Pathological changes in myocardial cells under electron microscope

**Fig. 4:**
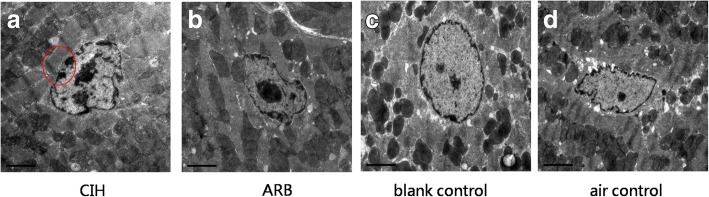
Pathological changes in myocardial cell nucleus under electron microscope. Ultrastructural examination was performed as described in materials and methods. **a** In CIH group, the myocardial nuclei were pyknotic, the heterochromatin clustered in the perinuclear region, which showed the apoptosis of myocardial cells. **b** In ARB group, the apoptosis of cardiomyocytes was rare. **c** & **d**. In blank and air control groups, the chromatin of myocardial nuclei was uniform and the nuclear membrane was intact. Magnification: 15000×, scale bar, 2 μm

**Fig. 5:**
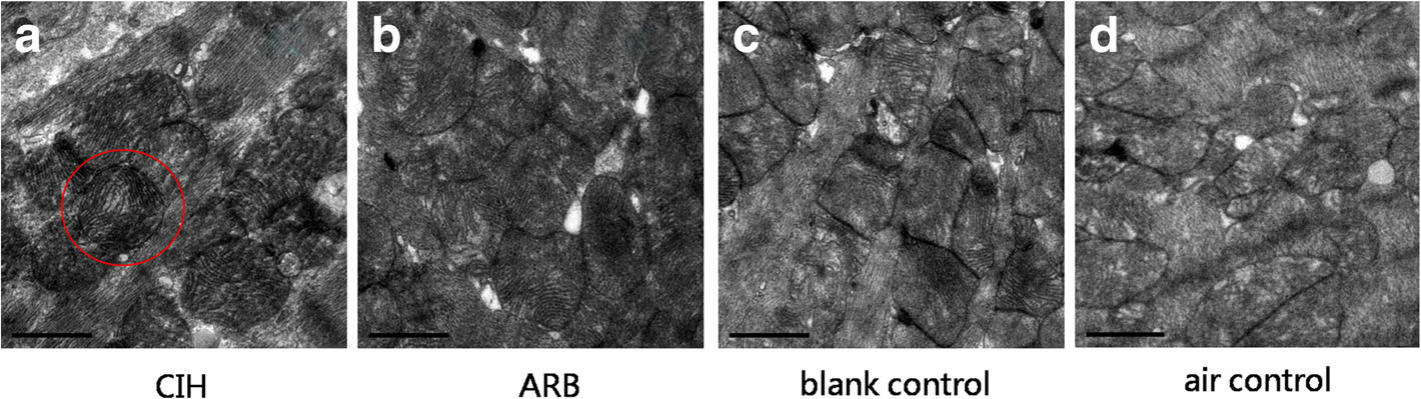
Pathological changes in myocardial cell mitochondria under electron microscope. Ultrastructural examination was performed as described in materials and methods. **a** In CIH group the number of mitochondria decreased, the size was abnormal, the mitochondrial membrane was incomplete, the number of cristae was reduced, and the arrangement was disorder. The cristae were broken, dissolved, destroyed, flocculent, and the electron density of matrix increases. **b** In ARB group, the quantity and size of the mitochondrial are normal and the membrane is intact. The electron density of the mitochondrial matrix increased the same as CIH group while the number of cristae was normal and well arranged. **c** & **d** In blank and air control groups, the quantity and size of the mitochondrial are normal and the membrane is intact. The mitochondrial cristae were filled with the entire cavity of the mitochondrion and were arranged in parallel, regular and abundant in number. Magnification: 40000×, scale bar, 1 μm

**Fig. 6:**
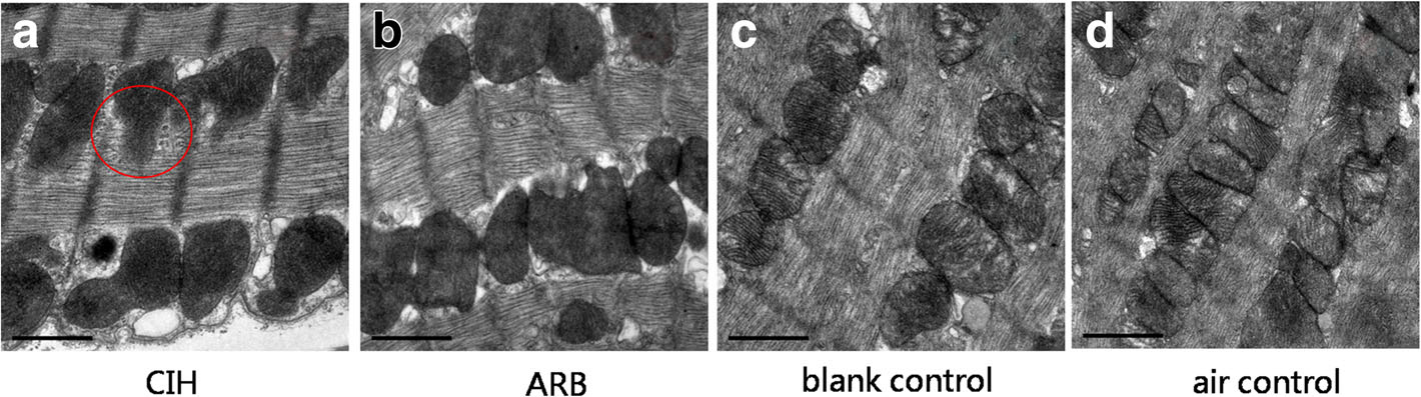
Pathological changes in myocardial cell mitochondria and myoneme under electron microscope. Ultrastructural examination was performed as described in materials and methods. **a** In CIH group, the number of mitochondria decreased, the size was unequal and abnormal, the mitochondrial membrane was incomplete, and the electron density of matrix increased. **b** In ARB group, the electron density of the mitochondrial matrix increased the same as CIH group while the quantity and size of the mitochondria were normal and the membrane was intact. **c** & **d**. In blank and air control groups, the electron density of the mitochondrial matrix was nomal, the quantity and size of the mitochondria were normal and the membrane was intact. The coarse and fine filaments were well arranged and each band was clearly visible in four experimental groups (**a**-**d**). Magnification: 40000×, scale bar, 1 μm

**Fig. 7:**
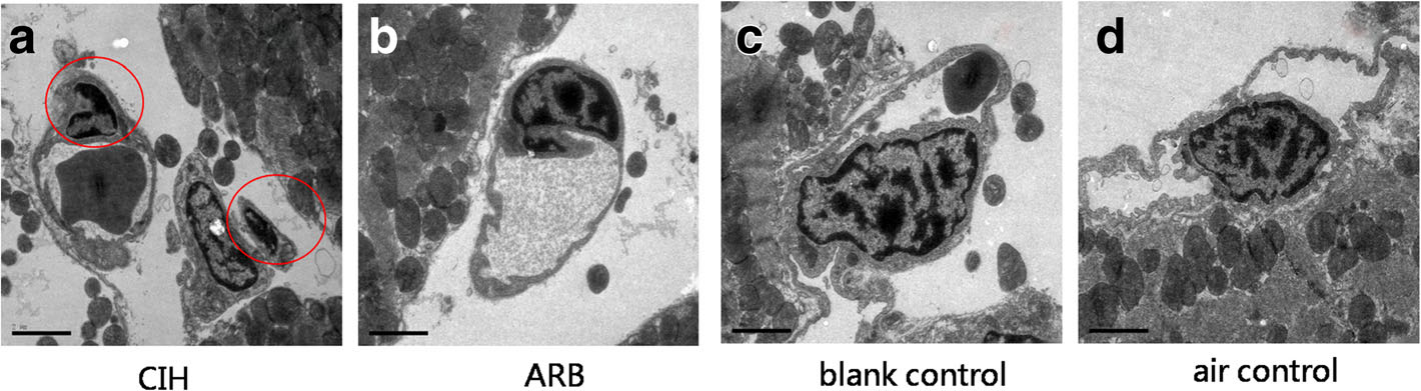
Pathological changes in capillary endothelial cells under electron microscope. Ultrastructural examination was performed as described in materials and methods. Increased capillary endothelial cell apoptosis was observed in CIH group (**a**), which was rarely seen in ARB group (**b**), **c** & **d**. In blank and air control groups the chromatin of capillary endothelial cell was uniform and the nuclear membrane was intact. Magnification: 15000×, scale bar, 2 μm

Next, we evaluated the pathological changes in myocardial cell and capillary endothelial cells in the four experimental groups under electron microscope. While in both the blank and air control groups the chromatin of myocardial nuclei was uniform and the nuclear membrane was intact, in CIH group the myocardial nuclei were pyknotic, the nucleolus dissolved and disappeared, and the heterochromatin clustered in the perinuclear region, which showed the apoptosis of myocardial cells in 8/8 CIH cases (Fig. 4a-d). Also, in CIH group, the number of mitochondria decreased, the size was abnormal, the mitochondrial membrane was incomplete, the number of cristae was reduced, and the arrangement was disordered, while in the blank and air control groups, the quantity and size of the mitochondrial are normal and the membrane is intact. The mitochondrial cristae filled the entire cavity of the mitochondrion and were arranged in parallel, regular and abundant in number (Fig. 5a-d). In addition, in CIH group, the cristae are broken, dissolved, destroyed, flocculent, as well as increased electron density of matrix increases in 7/8 CIH cases (Fig. 6a-d). The coarse and fine filaments were well arranged and each band was clearly visible in CIH group, blank and air control groups. Capillary endothelial cell nuclei were pyknotic, and the heterochromatin clustered in the perinuclear region which showed the apoptosis of capillary endothelial cell in 4/8 CIH cases (Fig. 7a-d) while no capillary endothelial cells were found apoptotic in the blank and air control groups.

The ultrastructural changes observed in the ARB group were in between those of the CIH group and the blank and air control groups. Visualization of cardiomyocyte and capillary endothelial cell apoptosis is rare under electron microscopy. The quantity and size of the mitochondria were normal with intact membranes. The electron density of the mitochondrial matrix increased similarly to that observed in the CIH group, however, the number of cristae was normal and well-arranged akin to that of the air and blank control groups. The changes under the electron microscope were closer to blank control and air control groups. Pathological changes were alleviated obviously in ARB group.

**Fig. 8:**
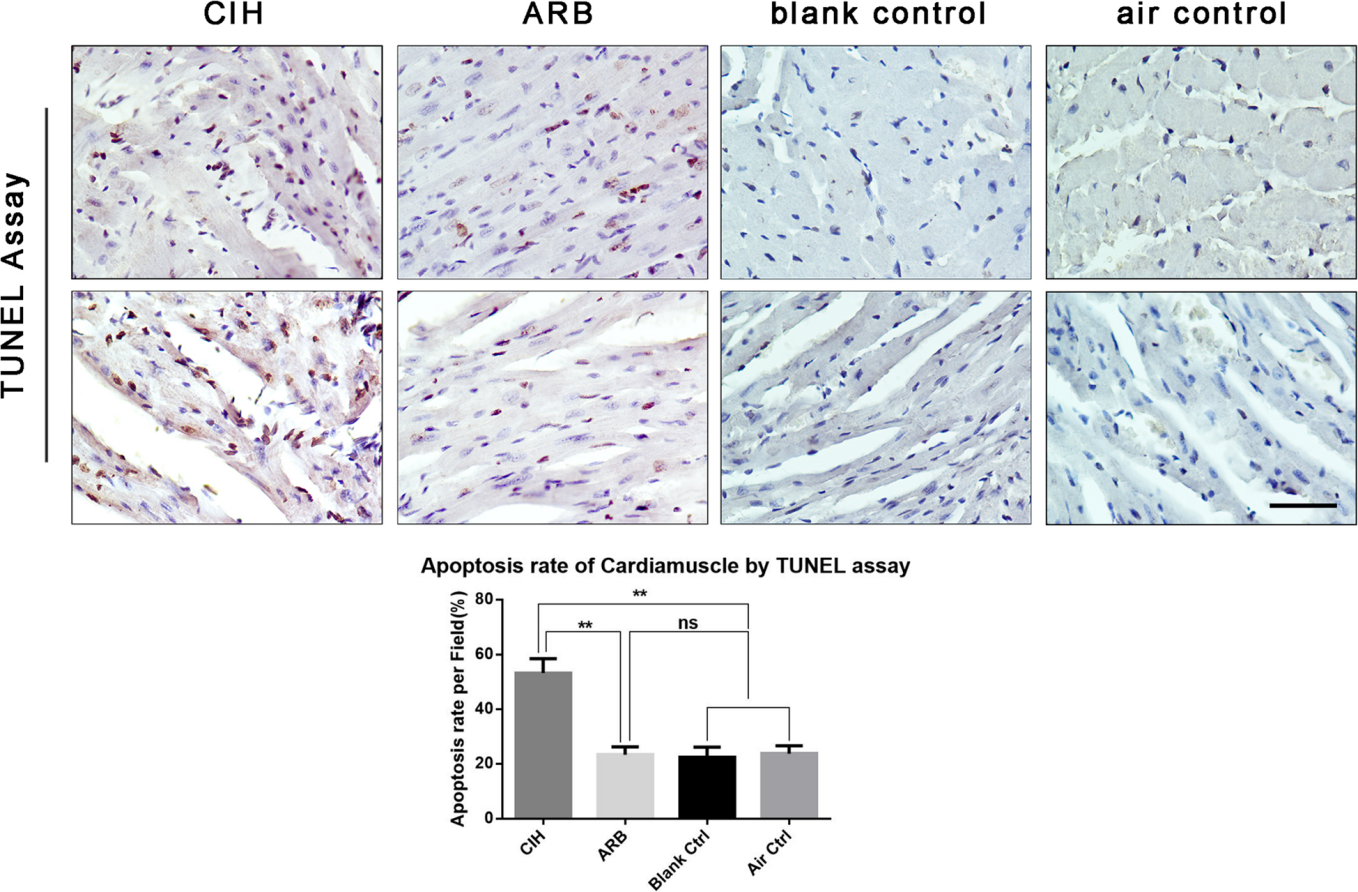
Apoptotic rate of myocardial cells in four experimental groups. TUNEL staining was performed as described in materials and methods. CIH group exhibited the highest apoptotic rate compared with the other three experimental groups. **p < 0.001 vs. CIH group. Magnification: 400×, scale bar, 100 μm

We also assessed apoptosis of myocardial cells of mice from these four groups with TUNEL assay. As shown in Fig. 8, while no significant difference in apoptotic rate of myocardial cells was observed between ARB group, blank control and air control groups, which are quantified and presented in Table 1. CIH had a significantly higher apoptotic rate compared with the other three experimental groups (^**^p < 0.001). Thus, CIH impairs myocardial cells structure and increases myocardial cells and capillary endothelial cells apoptosis while these changes can be reversed with TERT treatment.

## Discussion

SAS is closely linked to the development of cardiovascular disease, in which CIH plays an important role. In our experiment, each intermittent hypoxia cycle lasted approximately two minutes, mimicking 30 times intermittent hypoxia per hour, which was equivalent to severe SAS. Chen et al. [9] studied a CIH animal model and concluded that the lowest oxygen saturation was approximately 70%, equivalent to severe SAS. In another model, Song et al. [10] also demonstrated that, owing to the gas constant exchange in the box, there was no carbon dioxide accumulation. The partial pressure of carbon dioxide in the blood of mice had no correlation with the intermittent hypoxia cycle, which confirmed that the mice did not rebreathe. These findings eliminated the interference of carbon dioxide retention.

CIH can stimulate hypoxic chemoreceptors in the carotid body, thus activating the systemic sympathetic nervous system and RAS [2], but how CIH affects local RAS and subsequently contributes to the development of CVD remains unknown. In our study, we found that ACE expression was highest in the CIH group (Fig. 1a, b), suggesting that CIH could facilitate the expression of ACE in mouse cardiac myocytes in vivo. ACE is the key enzyme of RAS and participates in the systemic and local effects of RAS through endocrine, autocrine and paracrine secretion. The most prominent physiological activity of ACE is to cleave Ang I to Ang II and inactivate bradykinin. Ang II is the product of ACE [11, 12], and ACE can damage the cardiovascular system through production of Ang II. Our study also demonstrated that Ang II expression was higher in the CIH group (Fig. 2a, b) than in the blank control and air control groups (Fig. 2e-h), indicating that CIH induced the expression of ACE and its product Ang II which is potentially implicated in CIH-linked CVD. AngII can act on the angiotensin type 1 receptor, and increase inflammation, oxidative stress, apoptosis, and cardiac remodeling, ultimately leading to heart failure. [13] As Ang II can up-regulate the expression of ACE [14], we believe that there was a positive feedback between Ang II and ACE.

ACE 2 is the homolog of ACE, which shares 42% sequence homology with ACE. The main function of ACE 2 is to cleave Ang I to Ang (1–9) and Ang II to Ang (1–7). The catalytic activity of cleaving Ang II to Ang (1–7) is 400 times higher than cleaving Ang I to Ang (1–9) [15]. Therefore, the main product of ACE 2 is Ang (1–7). Ang (1–7) exhibits anti-RAS activity, as well as functioning as a vasodilatation and anti-proliferative agent, suggesting that ACE 2 is the key enzyme in balancing the vasoconstrictive and proliferative effects of Ang II, as well as the anti-proliferative effect of Ang (1–7). In our study, we observed higher expression of ACE 2 in the myocardium of mice from CIH group (Fig. 3a, b) than that from blank and air control groups (Fig. 3e-h). Since increased ACE 2 levels offer benefits to cardiac structure and function, we speculate that CIH-induced expression of ACE 2 was an adaptive response. Indeed, Zhang R [16] demonstrated that ACE 2 mRNA and protein expression increased in the early stages of hypoxia in pulmonary artery smooth muscle cells, but decreased at a later stage, which was accompanied by the accumulation of hypoxia inducible factor-1α (HIF-1α) and Ang II. Thus, it is possible that HIF-1α downregulates ACE 2 expression through Ang II. In our experiments, CIH lasted for 12 weeks, and ACE 2 expression remained higher in the CIH group than in blank and air control groups. Whether local accumulation of HIF-1α and Ang II at later hypoxic stages was responsible for the downregulation of ACE 2 remains unclear, and requires further study. This may include examining the correlation of the expression levels of myocardial ACE 2 with HIF1α and Ang II in hypoxic heart tissue for a prolonged CIH time more than 12 weeks.

In TUNEL assays, we also observed that myocardial cell apoptosis was the most severe in CIH group. Under electron microscopy, the apoptosis of cardiomyocyte and capillary endothelial cells were the most serious in CIH group. We also observed the ultrastructural changes of mitochondria and cristae, indicating that CIH can also cause mitochondrial damage. Chen et al. reported that the damage of CIH to mitochondria was related to myocardial apoptosis [17]. Mitochondrial damage is one of the primary mechanisms leading to apoptosis, and hyperactivity of RAS can produce apoptosis in early stages of cardiac disease [18]. Activated RAS can also exacerbate mitochondrial damage and increase apoptosis in endothelial cells, and mitochondria-ROS acts as a central regulator of RAS-mediated cell damage [19].We surmise that CIH can lead to myocardial damage by elevating the expression of ACE and Ang II, while the higher expression of ACE2 presents insufficiency of compensation.

Our study also showed that Ang II expression was highest in ARB group (Fig. 2c, d). ARBs can protect the cardiovascular system by blocking the activation of angiotensin type 1 receptor by Ang II, therefore, inhibit the negative feedback for the release of renin. Finally, ARBs increase renin, Ang I, Ang II and Ang (1–7) [20, 21]. Increased Ang II concentrations can interact with the angiotensin type 2 receptor. It is believed that this is mediated by bradykinin and nitric oxide (NO) [22], and the effect of this combination of mediators is both vasodilatory and anti-proliferative, which protects the cardiovascular system. Ang II has been shown to up-regulate the expression of ACE [14], which can be blocked by angiotensin type 1 receptor blocker (ARB) but not by angiotensin-2 receptor blockers, indicating that Ang II-induced expression of ACE was mainly achieved through angiotensin type 1 receptor- (AT-1) activation. In the present study, treatment with TERT, an ARB, reduced the level of ACE induced by CIH, which might alleviate the myocardial damage of CIH. Although this reduction did not reach statistical significance compared with the CIH group, maximal pharmacological effects of TERT were observed between four to eight treatment weeks, and we only treated mice with TERT for four weeks, which may account for this observation. Therefore, longer treatment with TERT is needed to further clarify if TERT could confer protection against CIH-induced cardiac injury via efficiently suppressing an increase in ACE.

In our study, we also observed that TERT treatment significantly elevated the expression of ACE 2 in the heart, suggesting that this may be one of the mechanisms by which TERT protects against hypoxia-induced heart injury. Previously, Koka et al. [14] showed that Ang II down-regulated ACE 2 expression in HK-2 cell line, which was blocked by the angiotensin type 1 receptor blocker losartan an extracellular signal-regulated kinase (ERK1/2) and p38 MAP kinase-specific antagonist, but not by the angiotensin type 2 receptor blocker PD123319, suggesting that Ang II suppressed ACE 2 expression through angiotensin type 1 receptor-ERK1/2 and p38 MAP kinase signaling. In the present study, Ang II expression in the hearts was increased in the CIH group, and this increase was further elevated by TERT treatment. These observations were in line with the TERT-induced increase in ACE 2. But increased Ang II expression did not suppress the expression of ACE 2 in mouse CIH hearts with TERT treatment, which was most likely due to the blockage of angiotensin type 1 receptor- by TERT.

Under electron microscopy and through TUNEL assay, we observed that the apoptosis of cardiomyocytes and capillary endothelial cells were rare in the ARB group. The ultrastructural changes of mitochondria and cristae induced by CIH were also alleviated by ARB treatment. We hypothesize that the blockade of the angiotensin type 1 receptor (AT1R) by ARB treatment can elevate the expression of ACE2, thus enhancing the protective effects of ACE 2 and alleviating the myocardial damage induced by ACE and AngII.

Furthermore, TERT, acting as an ARB, can block the effects of Ang II more thoroughly than ACEI, such as Ang II generated from chymotrypsin or other non-ACE pathways. Therefore, TERT may replace ACEI in long-term clinical use to avoid “AngII inhibition escape” phenomenon.

## Conclusions

In conclusion, we demonstrate that CIH induces the expression of ACE and Ang II in mouse hearts which may impair cardiovascular function. CIH also increases the cardiac expression of ACE2, which may be a compensatory protection mechanism against CIH. TERT treatment further increased ACE 2 levels in myocardial cells but decreased ACE level, through blocking the interaction between Ang II and AT-1 receptor, impeding the effects of Ang II, thus conferring cardiac protection against CIH.

## Abbreviations

ACE 2: angiotensin converting enzyme 2
ACE: angiotensin converting enzyme
Ang II: angiotensin II
ANOVA: analysis of variance
AT1R: Angiotensin type 1 receptor
CIH: Chronic intermittent hypoxia
CIH: chronic intermittent hypoxia
DAB: diaminobenzidine
MDA: malondialdehyde
RAS: renin-angiotensin system
SAS: sleep apnea syndromes
TERT’: telmisartan
TUNEL: TdT-mediated dUTP nick end labeling
